# Adiposity and carotid-intima media thickness in children and adolescents: a systematic review

**DOI:** 10.1186/s12887-015-0478-5

**Published:** 2015-10-16

**Authors:** Min Hae Park, Áine Skow, Sara De Matteis, Anthony S. Kessel, Sonia Saxena, Russell M. Viner, Sanjay Kinra

**Affiliations:** Department of Non-communicable Disease Epidemiology, London School of Hygiene & Tropical Medicine, Keppel Street, London, WC1E 7HT, UK; Department of Respiratory Epidemiology, Occupational Medicine and Public Health, NHLI, Imperial College London, London, UK; Faculty of Public Health and Policy, London School of Hygiene & Tropical Medicine, London, UK; Department of Primary Care and Public Health, Imperial College London, London, UK; Department of General and Adolescent Paediatrics, Institute of Child Health, University College London, London, UK

**Keywords:** Childhood obesity, Carotid intima-media thickness, Cardiovascular risk

## Abstract

**Background:**

Adiposity in childhood is associated with later cardiovascular disease (CVD), but it is unclear whether this relationship is independent of other risk factors experienced in later life, such as smoking and hypertension. Carotid-intima media thickness (cIMT) is a measure of subclinical atherosclerosis that may be used to assess CVD risk in young people. The aim of this study was to examine the relationship between adiposity and cIMT in children and adolescents.

**Methods:**

We searched Medline, Embase, Global Health, and CINAHL Plus electronic databases (1980–2014). Population-based observational studies that reported a measure of association between objectively-measured adiposity and cIMT in childhood were included in this review.

**Results:**

Twenty-two cross-sectional studies were included (*n* = 7,366 children and adolescents). Thirteen of nineteen studies conducted in adolescent populations (mean age ≥12 years, *n* = 5,986) reported positive associations between cIMT and adiposity measures (correlation coefficients 0.13 to 0.59). Three studies of pre-adolescent populations (*n* = 1,380) reported mixed evidence, two studies finding no evidence of a correlation, and one an inverse relationship between skinfolds and cIMT. Included studies did not report an adiposity threshold for subclinical atherosclerosis.

**Conclusions:**

Based on studies conducted mostly in Western Europe and the US, adiposity does not appear to be associated with cIMT in pre-adolescents, but may be associated in adolescents. If further studies confirm these findings, a focus on cardiovascular disease prevention efforts in pre-adolescence, before arterial changes have emerged, may be justified.

**Electronic supplementary material:**

The online version of this article (doi:10.1186/s12887-015-0478-5) contains supplementary material, which is available to authorized users.

## Background

A number of studies have reported positive associations between body mass index (BMI) in childhood and cardiovascular disease (CVD) risk factors, morbidity and mortality in adulthood [1–4]. However, childhood obesity tracks into adulthood [5], and where studies have been able to account for obesity in adulthood, the associations between childhood obesity and adult cardiovascular disease have been less convincing [6]. This raises questions about the appropriateness of directing cardiovascular disease prevention efforts towards overweight and obese children [7]. Furthermore, many of the other conventional risk factors for cardiovascular disease, such as smoking, alcohol intake, high serum cholesterol and blood pressure [8, 9], are more prevalent in overweight individuals than their lean counterparts. These other risk factors could explain the observed association between childhood obesity and future cardiovascular disease.

The standard method of accounting for potential alternative explanations in epidemiological studies (i.e. confounding) is to adjust for them in multiple variable regression models. However, this technique is strongly reliant on the accuracy with which these variables can be assessed. The difficulties of accurate variable measurement (e.g. assigning social position or assessing smoking, alcohol or dietary intake accurately from self-reports, and accounting for varying periods of exposure) make adjusted results prone to bias. An alternative analytical approach is to restrict the analyses to sub-groups that have little or no exposure to these risk factors. For cardiovascular disease, the prevalence of many of the conventional risk factors such as smoking, excessive alcohol intake, hypercholesterolemia, hypertension or diabetes, is negligible in children and low in adolescents [10–12], making them an ideal group in which to examine the independent contribution of childhood obesity to future CVD risk. Carotid intima media thickness (cIMT) is a measure of subclinical atherosclerosis that is a surrogate for future cardiovascular disease [13, 14]. A recent systematic review examined the relationship between BMI and cIMT [15], but reported only mean differences in cIMT by categorical weight status rather than the continuous relationships between measures of adiposity and cIMT, and the review was limited to studies in children aged 5–15 years.

We systematically reviewed the published evidence on the association between measures of adiposity in childhood or adolescence and cIMT. We limited our study to population-based samples, with a view to identifying any thresholds above which the potential effects of adiposity may manifest. We also sought evidence for variations in these associations by age, sex and ethnicity.

## Methods

We searched for published English-language studies of the association between measures of adiposity (including BMI, weight status, body fat percentage, waist circumference) and cIMT (reported as continuous or categorical measures) in childhood and adolescence. Medline, Embase, Global Health, and CINAHL Plus electronic databases were searched (1980-November Week 2, 2014) for relevant publications. Ethical approval was not sought for this study as it was a review of published literature already in the public domain; data were analysed as reported in the original publications.

The following search terms were used for Medline and modified for the other databases: 1. exp Obesity/ 2. exp Overweight/ 3. exp Body Mass Index/ 4. exp “Body Weights and Measures”/ 5. (obes$ or obesity).mp. 6. Overweight.mp. 7. (BMI or Body mass index or Body-mass-index or Weight for height or Weight-for-height).mp. 8. (Body fat or Body fat percent$ or Percent$ body fat or Fat mass or Adiposity).mp. 9. (Waist circumference or Waist measurement).mp. 10. or/1-9. 11. exp Child/ 12. exp Adolescent/13. exp Pediatrics/ 14. juvenile.mp. 15. child$.mp. 16. adolescen$.mp. 17. teen$.mp. 18. P?ediatric$.mp. 19. or/11-18. 20. exp Carotid Intima-Media Thickness/ 21. (Intima?media thickness or Carotid intima?media thickness or Arterial thickness or Arterial wall thickness).mp. 22. or/20-21. 23. 10 and 19 and 22. 24. Limit 23 to (English language and humans and yr = “1980 -Current”).

Observational studies were eligible for inclusion in the review, while case reports and abstracts were excluded. Studies were included if all of the following criteria were met: (1) they reported on the association between adiposity and cIMT in childhood or adolescence (mean age of population between 2 and 19 years), (2) both adiposity and cIMT were assessed using objective measures, (3) adiposity and cIMT were measured within 24 months of each other, (4) the study was of a community/population-based sample, and (5) sample size ≥100. Studies were excluded if any of the following criteria were met: (1) the study population received an intervention, (2) adiposity was measured in infancy (<2 years), (3) adiposity was assessed based on self- or parent-reported measures, (4) the study population was from an obesity/specialist clinic or hospital setting, or (5) a participant’s inclusion in the study was dependent upon the presence of a secondary comorbidity.

Two independent reviewers (MHP and SDM) screened titles and abstracts for an initial assessment of eligibility. After abstract screening, both reviewers reviewed full text articles to make final decisions on inclusion. Disagreements regarding eligibility were resolved after consultation with a third reviewer (AS).

Data were extracted in duplicate by MHP and SDM using a piloted form. For each study, the following information was recorded: study design, country and dates of study, measures of adiposity and health outcomes used, prevalence of overweight and obesity, characteristics of the study population (sample size, sex, age, ethnicity), cIMT measurement methods, measures of the association between adiposity and cIMT (e.g. regression coefficients, relative risks), and any additional analyses (e.g. adjustment for covariates, stratification by other characteristics). Given the heterogeneity of study populations and measures of effect used, data were synthesised in a narrative fashion.

## Results

**Fig. 1:**
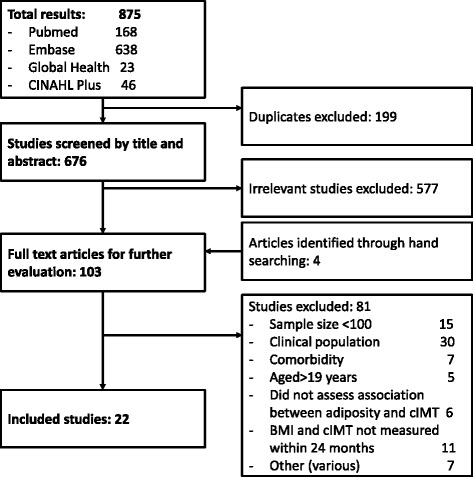
Flowchart of study selection process during systematic search

Of 676 abstracts screened, 22 studies were eligible for inclusion, representing 7,366 children and adolescents (Fig. 1). Thirteen studies were conducted in Europe [16–28], four in the United States [29–32], two in Turkey [33, 34], and one each in Chile [35], India [36] and South Korea [37]. With the exception of one French study [26], all studies were published within the last decade. Participants ranged in age from 4 to 24 years. Among those studies that reported the figure, the proportion of overweight and obese participants ranged from 11.6 % in Germany [16] to 74.0 % in Turkey [33]. The full descriptions of included studies, including methods used to measure cIMT, are presented in Table 1. The details of studies excluded during the full text review are listed in Additional file 1.

**Table 1:** Characteristics of studies included in review

Reference	Country	Population (n)	% overweight (incl. obese)	% female	Age range, years	cIMT measurement method
Arnaiz et al. 2010 [35]	Chile	Schools (103)	56	46.6	6−16	Ultrasonography; measurement location not reported; automated edge detection. Number of measurements not reported.
Böhm et al. 2009 [16]	Germany	Schools (267)	11.6	53.6	6−17	Ultrasonography; measurement proximal to carotid artery bifurcation; automated edge detection. Mean of 11 measurements used for analysis.
Casariu et al. 2011 [17]	Romania	Schools (100)	50	58.0	6−18	Ultrasonography; measurement at the common carotid artery near the bifurcation, during end diastole. Maximal thicknesses of the intima-media width measured to give three readings and the mean value was used for analysis.
Caserta et al. 2010 [18]	Italy	Primary schools (575)	31.1	49.9	11−13	Ultrasonography; measurement of far wall at 3 locations below bifurcation. Mean of these measurements used for analysis.
Croymans et al. 2010 [29]	United States	High schools (249)	15 % had BMI centile > 95th	67.5	15−18	Ultrasonography; images of the far wall of the common carotid artery taken from multiple angles; automated edge detection. Mean of these measurements used for analysis.
Dawson et al. 2009 [30]	United States	Offspring of population-based cohort (228 aged < 18)	NR	44.3	11−17	Ultrasonography; near and far wall of the left and right internal, bifurcation and common carotid arteries imaged at three angles; automated edge detection. The mean across angles used to obtain location-specific means; the average of these 12 measures used for analysis.
Doyon et al. 2013 [19]	Turkey, Germany, Sweden, Poland	Nonobese, nonhypertensive children (1,051)	Nonobese sample	53.2	6−18	Ultrasonography; cIMT was obtained either by 5 averaged measurements on each common carotid artery or semiautomatically using a digital image evaluation software, depending on the availability of the software package at each centre.
Elkiran 2013 [33]	Turkey	Schools (123)	74.0	54.5	11−15	Ultrasonography; measurement of left common carotid artery. Number of measurements not reported.
Geerts 2012 [20]	Netherlands	Population-based birth cohort (306)	NR	55.5	5	Ultrasonography (high resolution echo-tracking); measurement of right common carotid artery. Measurement repeated a maximum of four times.
Jourdan et al. 2005 [21]	Germany and Poland	Schools (247)	NR	51.4	10−20	Ultrasonography; far wall measured manually using the calliper method.
Kollias et al. 2013 [22]	Greece	Schools (448)	28.1 % overweight, 12.7 % obese	52.9	10−18	Ultrasonography; bilateral measurements at the point of maximum thickness on the far wall along a 1 cm section of each common carotid artery proximal to the carotid bulb; measurement using electronic calipers. Mean of 3–4 measurements for each side used for analysis.
Lamotte et al. 2013 [23]	France	Schools (319)	13.5 % overweight 3.4 % obese	57.7 %	12.5−17.5	Ultrasonography; bilateral assessment along 10 mm segment of common carotid arteries, ≥5 mm from the bifurcation; one hundred measurements on average on the far wall on each side; automated. Mean value of left and right measures used for analysis.
Lim et al. 2009 [37]	South Korea	High school (285)	13.3	48.4	14−17	Ultrasonography; bilateral measurements of near and far walls of common carotid arteries; Automated edge-detection. Maximum IMT value determined for each side and the average used for analysis.
Melo et al. 2014 [24]	Portugal	Schools (385)	28.3	50.9	11−13	Ultrasonography; measurement on far wall of right common carotid artery; Automated edge-detection. Number of measurements not reported.
Mittelman et al. 2010 [31]	United States	Schools and universities (599)	32.7	51.3	6−20	Ultrasonography; measurement on far walls of left common carotid artery 1 cm proximal to the bifurcation during 3 complete separate cardiac cycles; Automated edge-detection. Average reading of all 3 systolic and 3 diastolic frames used for analyses.
Osiniri et al. [25] 2012	Spain	Well child visits at primary care centres (135)	NR	53.3	NR, mean 7.1 ± 1.1	Ultrasonography; diastolic images obtained from far wall of the distal common carotid artery 1 cm from bifurcation. Averages of 5 measurements used for analyses.
Ozguven et al. 2010 [34]	Turkey	Schools (142)	49.3	55.6	13−18	Ultrasonography; measurement on far wall of left common carotid artery. Mean of at least four measurements taken ~10 mm proximal to bifurcation used for analyses.
Pandit et al. 2014 [36]	India	Private schools; routine health checks (250)	71.2	NR	6−17	Ultrasonography (echo-tracking); measurement at right common carotid artery; Automated edge-detection. Number of measurements not reported.
Sass et al. 1998 [26]	France	Population-based cohort (193)	NR	56.0	10−24 (mean 15.5)	Ultrasonography; bilateral measurement on ≥1 cm segment of carotid arteries at 3 cm proximal to the bifurcation. Average of 25–50 readings per measurement, with two measurements obtained per segment. Average of right and left measurements used for analysis.
Urbina et al. 2009 [32]	United States	318	43.4	62.3	10−24 (mean age 17.8)	Ultrasonography; bilateral measurements of three segments of carotid arteries. Trace technique to measure maximum thickness on right and left sides, and averaged for the common carotid artery, the bifurcation (carotid bulb), and the internal carotid artery.
Weghuber et al. 2013 [27]	Austria	Residents of Graz and Styria (104 subsample)	46.2	56.7	4−18	Ultrasonography; bilateral measurements of the bulbous near common carotid arteries. Maximal IMT recorded at each of the vessel segments and averaged for each side.
Whincup et al. 2012 [28]	United Kingdom	Primary schools (939)	NR	53	NR, mean 10.8 ± 0.4	Ultrasonography; bilateral measurement on far walls of common carotid arteries proximal to the carotid bifurcation. Three end-diastolic frames selected and analyzed for mean cIMT on each side. Mean of left- and right-sided readings used for analysis.

*BMI* body mass index, *NR* not reported, *cIMT* carotid intima-media thickness

### The association between adiposity and cIMT

Nineteen studies reported the relationship between adiposity and cIMT in adolescents or mixed populations of children and adolescents (mean age ≥12 years, *n* = 5,986). The other three studies were conducted in pre-adolescent populations (mean age <12 years, *n* = 1,380) [20, 25, 28].

#### Studies in mixed age or adolescent populations

Twelve studies of mixed age and adolescent-only samples reported on the correlation between measures of adiposity and cIMT (Table 2). Eight of these (sample sizes ranged from 100 to 1,051) found strong evidence of associations between adiposity measures (BMI, BMI-SDS, body fat percentage, body fat mass, waist circumference, hip circumference, waist to hip ratio) and cIMT, though in one study [16] the association was observed for girls only. After adjusting for other risk factors in multiple linear regression analyses, measures of adiposity remained an independent predictor of cIMT in six out of seven studies: one study found that BMI SDS was a predictor of age- and height-adjusted cIMT SDS [21]; another reported that after adjusting for ethnicity, systolic blood pressure, HDL-C, LDL-C and total cholesterol/HDL-C ratio, BMI z-score (*β* = 0.008, *p* < 0.0001) and waist circumference (*β* = 0.001, *p* = 0.0005) were predictive of cIMT in both sexes [31]; a third study showed that waist to hip ratio remained associated with cIMT after adjusting for age, sex, height, BMI, total cholesterol and blood pressure, but BMI did not [30]; another found BMI SDS to be associated with cIMT after adjusting for systolic blood pressure SDS (*β* = 0.14, *p* < 0.0001) [19]; in a stepwise multivariable analysis including age, sex, BMI, waist circumference, blood pressure, triglycerides to HDL-C ratio and glucose, waist circumference was found to be a predictor of cIMT (*β* = 0.001, *p* < 0.01), but BMI was not [22]; in the sixth study, after including waist circumference, BMI, body fat mass, trunk fat mass, sex and age in a multivariable model, waist circumference remained associated with cIMT (*β* = 0.31, *p* = 0.03), but the other measures of adiposity did not [24]. The seventh study found that after adjustment for total % body fat, blood pressure and age, BMI was no longer predictive of cIMT in either sex [16].

**Table 2:** Associations between adiposity and cIMT in children and adolescents, expressed as correlation coefficients

Reference	Age range, years	Adiposity measure	All participants		Females		Males	
			r	P	r	P	r	P
**Studies in mixed age or adolescent populations (mean age ≥12 years)**								
Böhm et al. 2009 [16]	6-17	BMI	-	-	**0.35**	<0.001	0.14	0.11
		Body fat %	-	-	**0.41**	<0.001	0.06	0.46
Casariu et al. 2011 [17]	6-18	BMI	**0.49**	<0.05	-	-	-	-
		Waist circumference	**0.59**	<0.05	-	-	-	-
		Hip circumference	**0.58**	<0.05	-	-	-	-
Croymans et al. 2010 [29]	15-18	BMI centile	0.08*	0.22*	-	-	-	-
Dawson et al. 2009 [30]	11-17	BMI	**0.173**	<0.05	-	-	-	-
		Waist/hip ratio	**0.310**	<0.001	-	-	-	-
Doyon et al. 2013 [19]	6-18	BMI	**0.13**	<0.001	-	-	-	-
		BMI SDS	**0.13**	<0.001	-	-	-	-
Jourdan et al. 2005 [21]	10-20	BMI	**0.25**	<0.001	-	-	-	-
		BMI SDS^a^	**0.41**	<0.001	-	-	-	-
Kollias et al. 2013 [22]	10-18	BMI Z-score	0.05	NS	-	-	-	-
		Waist circumference	**0.12**	<0.05	-	-	-	-
		Waist to hip ratio	**0.12**	<0.05	-	-	-	-
Lamotte et al. 2013 [23]	12-17	BMI	0.03	NS	−0.03	NS	0.09	NS
		BMI Z-score	0.04	NS	−0.002	NS	0.08	NS
		Fat mass	−0.04	NS	−0.06	NS	0.04	NS
		Waist circumference	0.04	NS	−0.07	NS	0.1	NS
		Waist/hip ratio	0.04	NS	−0.03	NS	0.02	NS
Lim et al. 2009 [37]	14-17	BMI	0.0499	0.402	-	-	-	-
		Waist circumference	−0.0215	0.718	-	-	-	-
Melo et al. 2014 [24]	11-13	BMI	**0.188**	<0.001	-	-	-	-
		Body fat mass by DXA	**0.153**	0.003	-	-	-	-
		Trunk fat mass by DXA	**0.155**	0.002	-	-	-	-
		Waist circumference	**0.221**	<0.001	-	-	-	-
Mittelman et al. 2010 [31]	6-20	BMI z-score	-	-	**0.34**	<0.001	**0.30**	<0.001
		Waist circumference	-	-	**0.33**	<0.001	**0.32**	<0.001
		Hip circumference	-	-	**0.27**	<0.001	**0.26**	<0.001
		Waist/hip ratio	-	-	**0.20**	0.005	**0.13**	0.0271
Sass et al. 1998 [26]	10-24	Fat mass (kg)	-	-	−0.029	NS	0.135	NS
		Body fat %	-	-	−0.057	NS	0.024	NS
		BMI	-	-	−0.033	NS	0.146	NS
		Waist/hip ratio	-	-	0.069	NS	−0.016	NS
**Studies in pre-adolescent populations (mean age <12 years)**								
Osiniri et al. 2012 [25]	Mean 7.1 ± 1.1	BMI z-score^b^	0.040	NS	-	-	-	-
		Body fat %	0.042	NS	-	-	-	-
		waist circumference^b^	0.048	NS	-	-	-	-

Coefficients in bold were statistically significant (*P* < 0.05)

*cIMT* carotid intima-media thickness, *BMI* body mass index kg/m^2^, *DXA* Dual-energy X-ray absorptiometry, *NS* not significant, *P*-value not reported, *SDS* standard deviation score

*r and *P* values obtained from the author

^a^Outcome was age- and height-specific cIMT standard deviation score

^b^Log transformed

The four studies that did not find strong evidence of a correlation between adiposity and cIMT (sample sizes ranged from 193 to 319) also assessed a wide range of adiposity measures, including BMI [23, 26, 37], BMI centile [29], fat mass [23, 26], waist circumference [23, 37], waist to hip ratio [23, 26] and body fat percentage [26].

Seven additional studies conducted in adolescents examined the relationship between weight category and cIMT values. Five out of these seven studies reported positive associations between weight category and cIMT (Table 3). Caserta et al. [18] reported that the prevalence of abnormal cIMT (defined as values above the 75th centile of the study population) was higher in obese adolescents than non-overweight adolescents (41.4 % versus 20.7 % among females, 43.4 % versus 28.3 % among males). Four studies reported that mean cIMT was higher in overweight and obese children than in normal weight children (Table 3) [27, 33, 34, 36], with the difference in cIMT between obese and normal weight groups ranging from 0.03 mm [36] to 0.2 mm [27].

**Table 3:** Other measures of associations between cIMT and adiposity measures in children and adolescents

Reference	Age range, years	Adiposity measure(s)	Measure of association		*P* value
**Studies in mixed age or adolescent populations (mean age ≥12 years)**					
			Mean cIMT (mm)		
Arnaiz 2010 [35]	6-16	Normal weight	0.50 ± 0.03		NR
		Overweight	0.49 ± 0.02		
		Obese	0.49 ± 0.03		
Elkiran 2013 [33]	11-15	Healthy weight	**0.36 ± 0.009**		0.001
		Overweight	**0.52 ± 0.008**		
		Obese	**0.53 ± 0.008** ^c^		
Ozguven et al. 2010 [34]	13-18	Normal weight	**0.51 ± 0.005**		<0.001
		Overweight	**0.57 ± 0.009**		
		Obese	**0.64 ± 0.007** ^d^		
Pandit et al. 2014 [36]	6-17	Normal weight	**0.31 ± 0.01**		<0.05
		Overweight/obese	**0.34 ± 0.01**		
Urbina et al. 2009 [32]	10-24	Lean	0.52 ± 0.08		NS
		Obese	0.50 ± 0.09		
Weghuber et al. 2013	4-18	Normal weight	**0.5 (95 % CI 0.4 to 0.6)**		<0.001
		Overweight/obese	**0.7 (95 % CI 0.6 to 0.7)**		
			cIMT >75th centile (%)		
Caserta et al. 2010 [18]	11-13		Males	Females	
		Non-overweight	28.3 %	20.7 %	<0.05
		Overweight	38.9 %	**34.8 %** ^a^	
		Obese	**43.4 %** ^a^	**41.4 %** ^a,b^	
**Studies in pre-adolescent populations (mean age <12 years)**					
			Mean cIMT (μm)		
Geerts et al. 2012 [20]	5	BMI tertiles			0.17
		First	380.4 ± 37.4		
		Second	387.4 ± 32.8		
		Third	389.9 ± 41.0		
			Change in cIMT (mm) per SD increase of adiposity measure		
Whincup et al. 2012 [28]	NR, mean 10.8 ± 0.4	Ponderal index^a^	−0.0007 (−0.0029 to 0.0015)		0.54
		Skinfolds^a^	−**0.0026 (−0.0048 to −0.0004)**		0.02
		Fat mass index^a^	−0.0019 (−0.0041 to 0.0003)		0.09

*Numbers in bold were statistically significant (P < 0.05)*

cIMT carotid intima-media thickness, *NR* not reported, *NS* not significant, *p*-value not reported, *CI* confidence interval, *SD* standard deviation

^a^Log transformed and adjusted for age, sex, ethnicity, observer, and month

^b^From Fisher exact test compared to non-overweight

^c^Overweight/obese compared to healthy weight

^d^From Kruskal-Wallis test

#### Studies in pre-adolescent populations

Three studies of pre-adolescents (mean age <12 years) did not find evidence of a positive association between cIMT and adiposity [20, 25, 28]. The largest of these was a UK study of school children with a mean age of 10.8 years (*n* = 939), which reported the absolute difference in cIMT for a 1 standard deviation increase in adiposity measures of ponderal index, fat mass index, and sum of skinfolds [28]; after adjusting for age, sex, ethnicity, observer, and month, there was no association between cIMT and either ponderal index or fat mass index, but sum of skinfolds was inversely associated with cIMT (Table 3). A Spanish study [25] found that in children aged 6–8 years, there was no correlation between cIMT and body fat percentage, BMI z-score, waist circumference, or visceral fat (Table 2). Similarly, a Dutch study of children aged 5 years showed that there was no association between BMI tertiles and cIMT [20].

### Factors affecting the association between adiposity and cIMT

Four studies presented results from analyses stratified by sex [16, 23, 26, 31]. In univariate analyses, Böhm et al. [16] observed a stronger correlation between cIMT and each adiposity measure (BMI and body fat %) in females than in males (Table 2), though these effects were attenuated to the null in multivariable analyses. In their analysis of a large US sample (*n* = 599), Mittelman et al. [31] found no difference in the effect of adiposity on cIMT by sex: the correlation coefficients for all reported adiposity measures were similar for males and females (Table 2). The other two studies reported negative correlation coefficients in girls and positive coefficients in boys, but none of these effects was statistically significant [23, 26]. None of the studies included in this review reported the relationship between adiposity and cIMT by ethnicity or age.

## Discussion

This review has shown there is a growing body of evidence for a positive relationship between adiposity and cIMT in adolescents, but not in younger children. The studies we included did not identify a threshold level of adiposity that led to increased cIMT, and there was little information available on whether the association between adiposity and cIMT varied by characteristics such as ethnicity, age or lifestyle behaviors.

Thirteen out of nineteen studies of adolescent populations (mean age ≥12 years) reported positive associations between cIMT and adiposity measures, including measures of weight-for-height (BMI), body composition (fat mass, body fat percentage), and fat distribution (waist and hip circumferences and their ratio). Comparing studies that found an association between adiposity and cIMT with studies that did not, there was no clear pattern according to the measures of adiposity used or study location, but there may have been differences in sample characteristics, notably the prevalence of overweight and obesity. Three of the four studies that did not find evidence of a correlation between adiposity and cIMT were conducted in relatively lean populations in France [23, 26] and South Korea [37], with prevalence of overweight and obesity <17 %, compared to 30-60 % in the majority of studies that showed a positive correlation between adiposity and cIMT, although positive correlations were also found in samples with low prevalence of overweight [16] and no obese participants [19]. The fourth study examined data from three diverse school populations: a predominantly Hispanic (94 %) and female (78 %) student body, a mixed-ethnicity school, and a conservative religious school (Seventh Day Adventist [SDA]) with a majority of female (72 %) students [29]. Pooling the results from these populations may have masked important differences in risk profiles, as SDA students were significantly leaner than other students and Hispanic students had significantly lower mean cIMT values. Analyses were not stratified by school, therefore the potential impact of ethnicity or lifestyle (Seventh Day Adventists follow a primarily vegetarian diet and abstain from alcohol, tobacco, and caffeinated drinks [38]) on the effect of adiposity on cIMT could not be assessed.

Of the three studies of younger, pre-adolescent children, a large UK study of children with mean age 10.8 years reported mixed associations between cIMT and three measures of adiposity [28], while studies in younger Spanish and Dutch children found no strong evidence of an association [20, 25]. These results, when considered together with the other findings of this review, suggest that thickening of the carotid artery with adiposity may only become detectable in later childhood and adolescence. This is consistent with the age-related transition from aortic fatty streaks to atherosclerotic lesions [39], and could also indicate that exposure to excess adiposity (or some other risk factor associated with overweight) accelerates these age-related arterial changes [40]. Recent evidence suggests that duration, more than degree, of obesity is an important factor in the onset of CVD [41]. It may therefore be the case that a minimum duration of exposure to overweight is needed before atherosclerosis is detectable, and this is unlikely to occur in pre-adolescence. Another possible explanation is that metabolic complications associated with obesity such as insulin resistance, which become manifest with longer exposure to excess weight, may explain changes in cIMT rather than adiposity itself [42]. The physiological processes underlying the observed association in adolescents could not be ascertained from this review, but disentangling the independent and synergistic effects of age, obesity and other risk factors should be a focus of future research.

The studies included in our review reported correlation coefficients that suggest that the association between adiposity and cIMT is linear, though few studies explicitly characterized the nature of the relationship. None of the studies reported a weight threshold for atherosclerosis at the studied levels of BMI; most of the studies were conducted in high income, western populations with relatively high prevalence of childhood overweight, therefore potential threshold effects at high levels of adiposity could have been assessed. A handful of studies conducted in populations with low prevalence of overweight and obesity did not examine threshold effects at low levels of adiposity. Further studies in populations that cover a wider range of BMI may be informative, as would those that describe in greater detail the nature (shape) of the association between adiposity and cIMT in young people. Studies that assess the effect of other risk factors on the relationship between adiposity and cIMT are also lacking; such studies may help to identify subgroups of the population that are at increased risk of arterial changes at any given level of adiposity, who may be targets for cardiovascular disease prevention interventions.

Our review expands on the findings of a recent meta-analysis, which reported that obese children have higher mean cIMT than non-obese children [15]. The meta-analysis included five studies, including two which we excluded because they were conducted in clinic-based populations [43, 44]. Our review included several recent studies that were published since the meta-analysis was conducted, and also included studies covering a wider age range (the meta-analysis included study populations aged 5 to 15 years), which enabled disaggregated assessment of studies conducted in pre- and post-adolescent populations.

One limitation of this review is that the finding of no association between adiposity and cIMT in younger children is based on only three studies. Furthermore, a meta-analysis of results was not conducted due to the heterogeneity of populations and adiposity measures used in the included studies. Additionally, we chose to use cIMT as a proxy for CVD risk in young people, despite the fact that there are few cardiovascular events in this age group. Also, variations in cIMT measurement methods may have affected our results, including the number and location of measurements, and whether measurements were manual or automated.

## Conclusions

Based on studies conducted mostly in Western Europe and the US, our review has shown that adiposity is positively correlated with cIMT in adolescents, but not in younger children. Studies are needed to confirm these findings, but if these relationships are consistently demonstrated in future research there may be justification for cardiovascular disease prevention efforts in overweight children that begin before adolescence, when arterial changes have yet to emerge.

## Availability of supporting data

For information about access to data extraction forms researchers should contact the corresponding author.

## Additional files

Additional file 1:
**Table of studies excluded from review.** (PDF 221 kb) Additional file 2:
**PRISMA checklist.** (DOC 63 kb)

## Abbreviations

BMI SDS: Body mass index standard deviation score
cIMT: Carotid-intima media thickness
CVD: Cardiovascular disease
