# Supplementary material for: Adiposity and carotid-intima media thickness in children and adolescents: a systematic review
Source: BMC Pediatr. 2015 Oct 16;15:161. doi: 10.1186/s12887-015-0478-5 (PMC4609088; doi:10.1186/s12887-015-0478-5)
Supplement: Additional file 1: — Table of studies excluded from review. (PDF 221 kb) [file 12887_2015_478_MOESM1_ESM.pdf]

Table S1: Summary of excluded studies

Reference	Main reason(s) for exclusion
Abaci A, Tasclar ME, Sartas T, Yozgat Y, Yesilkaya E, Kilic A, Okutan V, Lenk MK. Threshold value of subepicardial adipose tissue to detect insulin resistance in obese children. *International Journal of Obesity* 2009; 33: 440-46.	Sample size < 100; clinic population
Abdelghaffar S, El Amir M, El Hadidi A, El Mougi F. Carotid intima-media thickness: An index for subclinical atherosclerosis in type 1 diabetes. *Journal of Tropical Pediatrics* 2006; 52: 39-45.	Sample size < 100; clinic population
Alpsoy S, Akyuz A, Akkoyun DC, Nalbantoglu B, Topcu B, Donma MM. Effect of obesity on endothelial function and subclinical atherosclerosis in children<br>Cocuklarda obezitenin endotel fonksiyonu ve subklinik ateroskleroz uzerine etkisi. *European Journal of General Medicine.* 2014;11(3):141-7.	Clinic population
Aydin M, Bulur S, Alemdar R, Yalcin S, Turker Y, Basar C, et al. The impact of metabolic syndrome on carotid intima media thickness. *European Review for Medical & Pharmacological Sciences*. 2013;17(17):2295-301.	Adult population
Casariu ED, Virgolici B, Lixandru D, Stancu M, Greabu M, Totan A, et al. Relations for apoB/apoA-I ratio with different cardiovascular risk factors, in obese children. Romanian Journal of Biophysics. 2012;22(2):83-94.	Sample size <100
Chen LH, Zhu WF, Liang L, Yang XZ, Wang CL, Zhu YR, et al. Relationship between glycated haemoglobin and subclinical atherosclerosis in obese children and adolescents. Archives of Disease in Childhood. 2014;99(1):39-45.	Clinic population
Cheraghi N, Dai H, Raghuveer G. Vitamin D deficiency is associated with atherosclerosis-promoting risk factor clustering but not vascular damage in children. Medical Science Monitor. 2012;18(12):CR687-92.	Did not assess association between adiposity and cIMT
Chowdhury SM, Henshaw MH, Friedman B, Saul JP, Shirali GS, Carter J, et al. Lean body mass may explain apparent racial differences in carotid intima-media thickness in obese children. Journal of the American Society of Echocardiography. 2014;27(5):561-7.	Clinic population
Ciccone MM, Miniello V, Marchioli R, Scicchitano P, Cortese F, Palumbo V, et al. Morphological and functional vascular changes induced by childhood obesity. European Journal of Cardiovascular Prevention and Rehabilitation. 2011;18(6):831-5.	Sample size <100
Dangardt F, Chen Y, Berggren K, Osika W, Friberg P. Increased Rate of Arterial Stiffening with Obesity in Adolescents: A Five-Year Follow-Up Study. PLoS ONE. 2013;8(2).	Sample size <100
Daniels SR, Pratt CA, Hayman LL. Reduction of risk for cardiovascular disease in children and adolescents. Circulation 2011; 124: 1673-86.	Editorial report
Demircioglu F, Kocyigit A, Arslan N, Cakmakci H, Hizli S, Sedat AT. Intima-media thickness of carotid artery and susceptibility to atherosclerosis in obese children with nonalcoholic fatty liver disease. Journal of Pediatric Gastroenterology and Nutrition. 2008;47(1):68-75.	Study participation dependent on the presence of a secondary comorbidity
Erkocoglu M, Ozon ZA, Gocmen R, Alikasifoglu A, Gonc N, Kandemir N. Carotid intima media thickness in adolescents with increased risk for atherosclerosis. Turkish Journal of Pediatrics. 2013;55(5):510-8.	Study participation dependent on the presence of a secondary comorbidity

**Reference**	**Main reason(s) for exclusion**
Fang J, Zhang JP, Luo CX, Yu XM, Lv LQ. Carotid intima-media thickness in childhood and adolescent obesity relations to abdominal obesity, high triglyceride level and insulin resistance. International Journal of Medical Sciences. 2010;7(5):278-83.	Clinic population
Ford ES. Ideal cardiovascular health: Start young, finish strong. *Circulation* 2012; 125: 1955-57.	Editorial report
Freedman DS, Patel DA, Srinivasan SR, Chen W, Tang R, Bond MG, Berenson GS. The contribution of childhood obesity to adult carotid intima-media thickness: The Bogalusa Heart Study. *International Journal of Obesity* 2008; 32: 749-56.	Adiposity and cIMT not measured in childhood; adiposity and cIMT not measured within 24 months
Frontini MG, Srinivasan SR, Xu J, Tang R, Bond MG, Berenson GS. Usefulness of childhood non-high density lipoprotein cholesterol levels versus other lipoprotein measures in predicting adult subclinical atherosclerosis: The bogalusa heart study. *Pediatrics* 2008; 121: 924-29.	cIMT not measured in childhood
Ge W, Parvez F, Wu F, Islam T, Ahmed A, Shaheen I, et al. Association between anthropometric measures of obesity and subclinical atherosclerosis in Bangladesh. Atherosclerosis. 2014;232(1):234-41.	Adult population
Giannini C, de Giorgis T, Scarinci A, Ciampani M, Marcovecchio ML, Chiarelli F, et al. Obese related effects of inflammatory markers and insulin resistance on increased carotid intima media thickness in pre-pubertal children. Atherosclerosis. 2008;197(1):448-56.	Clinic population
Giannini C, de Giorgis T, Scarinci A, Cataldo I, Marcovecchio ML, Chiarelli F, Mohn A. Increased carotid intima-media thickness in pre-pubertal children with constitutional leanness and severe obesity: The speculative role of insulin sensitivity, oxidant status, and chronic inflammation. *European Journal of Endocrinology* 2009; 161: 73-80.	Clinic population
Gilardini L, Pasqualinotto L, Di Matteo S, Caffetto K, Croci M, Girola A, et al. Factors associated with early atherosclerosis and arterial calcifications in young subjects with a benign phenotype of obesity. Obesity. 2011;19(8):1684-9.	Clinic population
Gonzalez-Enriquez GV, Rubio-Benitez MI, Garcia-Gallegos V, Portilla-de Buen E, Troyo-Sanroman R, Leal-Cortes CA. Contribution of TNF-308A and CCL2-2518A to Carotid Intima-Media Thickness in Obese Mexican Children and Adolescents. Archives of Medical Research. 2008;39(8):753-9.	Clinic population
Gu W, Huang Y, Zhang Y, Hong J, Liu Y, Zhan W, et al. Adolescents and young adults with newly diagnosed Type 2 diabetes demonstrate greater carotid intima-media thickness than those with Type 1 diabetes. Diabetic Medicine. 2014;31(1):84-91.	Clinic population
Hacihamdioglu B, Okutan V, Yozgat Y, Yildirim D, Kocaoglu M, Lenk MK, et al. Abdominal obesity is an independent risk factor for increased carotid intima- media thickness in obese children. Turkish Journal of Pediatrics. 2011;53(1):48-54.	Clinic population
Huang K, Zou CC, Yang XZ, Chen XQ, Liang L. Carotid intima-media thickness and serum endothelial marker levels in obese children with metabolic syndrome. Archives of Pediatrics and Adolescent Medicine. 2010;164(9):846-51.	Clinic population

Reference	Main reason(s) for exclusion
Iannuzzi A, Licenziati MR, Acampora C, Salvatore V, Auriemma L, Romano ML, Panico S, Rubba P, Trevisan M. Increased carotid intima-media thickness and stiffness in obese children. *Diabetes Care* 2004; 27: 2506-08.	Clinic population
Juonala M, Magnussen CG, Venn A, Dwyer T, Burns TL, Davis PH, Chen W, Srinivasan SR, Daniels SR, Kahonen M, Laitinen T, Taittonen L, Berenson GS, Viikari J, Raitakari OT. Influence of age on associations between childhood risk factors and carotid intima-media thickness in adulthood: The cardiovascular risk in young finns study, the childhood determinants of adult health study, the bogalusa heart study, and the muscatine study for the international childhood cardiovascular cohort (i3C) consortium. *Circulation* 2010; 122: 2514-20.	cIMT not measured in childhood
Juonala M, Viikari JSA, Kahonen M, Taittonen L, Laitinen T, Hutri-Kahonen N, Lehtimaki T, Jula A, Pietikainen M, Jokinen E, Telama R, Rasanen L, Mikkila V, Helenius H, Kivimaki M, Raitakari OT. Life-time risk factors and progression of carotid atherosclerosis in young adults: The Cardiovascular Risk in Young Finns study. *European Heart Journal* 2010; 31: 1745-51.	cIMT not measured in childhood
Kandil ME, Anwar GM, Fatouh A, Salama N, Ahmed A, Elabd E, et al. Relation between serum homocysteine and carotid intima-media thickness in obese egyptian children. Journal of Clinical and Basic Cardiology. 2010;13(1-4):8-11.	Clinic population
Kelishadi R, Cook SR, Amra B, Adibi A. Factors associated with insulin resistance and non-alcoholic fatty liver disease among youths. *Atherosclerosis* 2009; 204: 538-43.	Clinic population; study participation dependent on the presence of a secondary comorbidity
Khadilkar AV, Chiplonkar SA, Pandit DS, Kinare AS, Khadilkar VV. Metabolic risk factors and arterial stiffness in Indian children of parents with metabolic syndrome. Journal of the American College of Nutrition. 2012;31(1):54-62.	Did not assess association between adiposity and cIMT
Kivimäki M, Davey Smith G, Juonala M, Ferrie JE, Keltikangas-Järvinen L, Elovainio M, Pulkki-Råback L, Vahtera J, Leino M, Viikari JSA, Raitakari OT. Socioeconomic position in childhood and adult cardiovascular risk factors, vascular structure, and function: cardiovascular risk in young Finns study. *Heart* 2006; 92: 474-80.	cIMT not measured in childhood
Knoflach M, Kiechl S, Penz D, Zangerle A, Schmidauer C, Rossmann A, Shingh M, Spallek R, Griesmacher A, Bernhard D, Robatscher P, Buchberger W, Draxl W, Willeit J, Wick G. Cardiovascular Risk Factors and Atherosclerosis in Young Women: Atherosclerosis Risk Factors in Female Youngsters (ARFY Study). *Stroke* 2009; 40: 1063-69.	Adiposity and cIMT not measured in childhood
Kocyigit A, Dogan M, Yilmaz I, Caglar M, Hatipoglu C, Kocyigit F, et al. Relation of age and sex with carotid intima media thickness in healthy children. Turkish Journal of Medical Sciences. 2014;44(3):422-6.	Sample size <100
Koopman LP, McCrindle BW, Slorach C, Chahal N, Hui W, Sarkola T, et al. Interaction between myocardial and vascular changes in obese children: a pilot study. Journal of the American Society of Echocardiography. 2012;25(4):401-10.e1.	Clinic sample; sample size <100

Reference	Main reason(s) for exclusion
Laitinen T, Laitinen TT, Pahkala K, Magnussen CG, Viikari JSA, Oikonen M, Taittonen L, Mikkila V, Jokinen E, Hutri-Kahonen N, Kahonen M, Lehtimaki T, Raitakari OT, Juonala M. Ideal cardiovascular health in childhood and cardiometabolic outcomes in adulthood: The cardiovascular risk in young Finns study. *Circulation* 2012; 125: 1971-78.	Adiposity and cIMT not measured in childhood; adiposity and cIMT not measured within 24 months
Le J, Zhang D, Menees S, Chen J, Raghuveer G. Vascular age is advanced in children with atherosclerosis-promoting risk factors. Circulation: Cardiovascular Imaging. 2010;3(1):8-14.	Study participation dependent on the presence of a secondary comorbidity
Leite A, Santos A, Monteiro M, Gomes L, Veloso M, Costa M. Impact of overweight and obesity in carotid intima-media thickness of portuguese adolescents. Acta Paediatrica. 2012;101(3):e115-21.	Clinic population
Lin HF, Liu CK, Liao YC, Lin RT, Chen CS, Juo SHH. The risk of the metabolic syndrome on carotid thickness and stiffness: Sex and age specific effects. *Atherosclerosis* 2010; 210: 155-59.	Adiposity and cIMT not measured in childhood; adiposity and cIMT not measured within 24 months
Litwin M, Sladowska J, Syczewska M, Niemirska A, Daszkowska J, Antoniewicz J, Wierzbicka A, Wawer ZT. Different BMI cardiovascular risk thresholds as markers of organ damage and metabolic syndrome in primary hypertension. *Pediatric Nephrology* 2008; 23: 787-96.	Clinic population; study participation dependent on the presence of a secondary comorbidity
Magnussen CG, Venn A, Thomson R, Juonala M, Srinivasan SR, Viikari JSA, Berenson GS, Dwyer T, Raitakari OT. The Association of Pediatric Low- and High-Density Lipoprotein Cholesterol Dyslipidemia Classifications and Change in Dyslipidemia Status With Carotid Intima-Media Thickness in Adulthood. Evidence From the Cardiovascular Risk in Young Finns Study, the Bogalusa Heart Study, and the CDAH (Childhood Determinants of Adult Health) Study. *Journal of the American College of Cardiology* 2009; 53: 860-69.	Adiposity and cIMT not measured in childhood; adiposity and cIMT not measured within 24 months
Magnussen CG, Koskinen J, Chen W, Thomson R, Schmidt MD, Srinivasan SR, Kivimäki M, Mattsson N, Kähönen M, Laitinen T, Taittonen L, Rönnemaa T, Viikari JS, Berenson GS, Juonala M, Raitakari OT. Pediatric metabolic syndrome predicts adulthood metabolic syndrome, subclinical atherosclerosis, and type 2 diabetes mellitus but is no better than body mass index alone: the Bogalusa Heart Study and the cardiovascular risk in young Finns study. *Circulation* 2010; 122: 1604-11.	Adiposity and cIMT not measured in childhood; adiposity and cIMT not measured within 24 months
Maple-Brown LJ, Cunningham J, Barry RE, Leylsey L, O'Rourke MF, Celermajer DS, O'Dea K. Impact of dyslipidaemia on arterial structure and function in urban Indigenous Australians. *Atherosclerosis* 2009; 202: 248-54.	Sample size < 100; adiposity and cIMT not measured in childhood; adiposity and cIMT not measured within 24 months
Miettinen TA, Gylling H, Hallikainen M, Juonala M, Rasanen L, Viikari J, Raitakari OT. Relation of non-cholesterol sterols to coronary risk factors and carotid intima-media thickness: The Cardiovascular Risk in Young Finns Study. *Atherosclerosis* 2010; 209: 592-97.	Adiposity and cIMT not measured within 24 months
Mimoun E, Aggoun Y, Pousset M, Dubern B, Bougle D, Girardet JP, et al. Association of Arterial Stiffness and Endothelial Dysfunction with Metabolic Syndrome in Obese Children. Journal of Pediatrics. 2008;153(1):65-70.e1.	Clinic population

Reference	Main reason(s) for exclusion
Miniello VL, Faienza MF, Scicchitano P, Cortese F, Gesualdo M, Zito A, et al. Insulin resistance and endothelial function in children and adolescents. International Journal of Cardiology. 2014;174(2):343-7.	Clinic population
Morrison KM, Dyal L, Conner W, Helden E, Newkirk L, Yusuf S, Lonn E. Cardiovascular risk factors and non-invasive assessment of subclinical atherosclerosis in youth. *Atherosclerosis* 2010; 208: 501-05.	Clinic population
Neuman G, Shavit I, Aronson D, Lorber A, Gaitini D, Onn R, et al. Cross-sectional analysis of cardiovascular risk factors in children with parental history of premature ischemic heart disease. Pediatric Cardiology. 2011;32(5):628-33.	Study participation dependent on the presence of a secondary comorbidity
Niu L, Zhang Y, Qian M, Meng L, Xiao Y, Wang Y, et al. Impact of multiple cardiovascular risk factors on carotid intima-media thickness and elasticity. PLoS ONE [Electronic Resource]. 2013;8(7):e67809.	Adult population
Ozcetin M, Celikyay ZR, Celik A, Yilmaz R, Yerli Y, Erkorkmaz U. The importance of carotid artery stiffness and increased intima-media thickness in obese children. South African Medical Journal Suid-Afrikaanse Tydskrif Vir Geneeskunde. 2012;102(5):295-9.	Sample size<100
Pacifico L, Bonci E, Andreoli G, Romaggioli S, Di Miscio R, Lombardo CV, et al. Association of serum triglyceride-to-HDL cholesterol ratio with carotid artery intima-media thickness, insulin resistance and nonalcoholic fatty liver disease in children and adolescents. Nutrition, Metabolism and Cardiovascular Diseases. 2014;24(7):737-43.	Did not report association between adiposity and cIMT
Pahkala K, Hietalampi H, Laitinen TT, Viikari JSA, Ronnemaa T, Niinikoski H, et al. Ideal cardiovascular health in adolescence effect of lifestyle intervention and association with vascular intima-media thickness and elasticity (the special turku coronary risk factor intervention project for children [STRIP] Study). Circulation. 2013;127(21):2088-96.	Did not report association between adiposity and cIMT
Pandit D, Chiplonkar S, Khadilkar A, Kinare A, Khadilkar V. Efficacy of a continuous metabolic syndrome score in Indian children for detecting subclinical atherosclerotic risk. International Journal of Obesity 2011; 35: 1318-24.	Did not report association between adiposity and cIMT
Pinto CS, Lana JM, Gabbay MA, de Sa JR, Dib SA. HDL cholesterol levels and weight are the main determinants of subclinical atherosclerosis in the young with type 1 diabetes and suitable glycaemic control. Diabetes & Vascular Disease Research. 2014;11(2):125-8.	Study participation dependent on the presence of a secondary comorbidity
Rathsman B, Rosfors S, Sjoholm A, Nystrom T. Early signs of atherosclerosis are associated with insulin resistance in non-obese adolescent and young adults with type 1 diabetes. Cardiovascular Diabetology. 2012;11:145.	Study participation dependent on the presence of a secondary comorbidity
Reinehr T, Wunsch R, Putter C, Scherag A. Relationship between carotid intima-media thickness and metabolic syndrome in adolescents. Journal of Pediatrics. 2013;163(2):327-32.	Clinic population
Rerkasem K, Wongthanee A, Rerkasem A, Chiowanich P, Sritara P, Pruenglampoo S, et al. Intrauterine nutrition and carotid intimal media thickness in young Thai adults. Asia Pacific Journal of Clinical Nutrition. 2012;21(2):247-52.	Adiposity and cIMT not measured in childhood
Sarkola T, Abadilla AA, Chahal N, Jaeggi E, McCrindle BW. Feasibility of very-high resolution ultrasound to assess elastic and muscular arterial wall morphology in adolescents attending an outpatient clinic for obesity and lipid abnormalities. Atherosclerosis. 2011;219(2):610-5.	Clinic population

**Reference**	**Main reason(s) for exclusion**
Sarkola T, Manlhiot C, Slorach C, Bradley TJ, Hui W, Mertens L, et al. Evolution of the arterial structure and function from infancy to adolescence is related to anthropometric and blood pressure changes. Arteriosclerosis, Thrombosis & Vascular Biology. 2012;32(10):2516-24.	Body surface area assessed as main exposure variable
Schiel R, Beltschikow W, Radón S, Kramer G, Perenthaler T, Stein G. Increased carotid intima-media thickness and associations with cardiovascular risk factors in obese and overweight children and adolescents. European journal of medical research. 2007;12(10):503-8.	Clinic population
Scuteri A, Orru M, Morrell C, Piras MG, Taub D, Schlessinger D, et al. Independent and additive effects of cytokine patterns and the metabolic syndrome on arterial aging in the SardiNIA Study. Atherosclerosis. 2011;215(2):459-64.	Mean age > 19 years
Scuteri A, Orru M, Morrell CH, Tarasov K, Schlessinger D, Uda M, Lakatta EG. Associations of large artery structure and function with adiposity: effects of age, gender, and hypertension. The SardiNIA Study. *Atherosclerosis* 2012; 221: 189-97.	Mean age > 19 years
Shah AS, Dolan LM, Gao Z, Kimball TR, Urbina EM. Clustering of risk factors: A simple method of detecting cardiovascular disease in youth. *Pediatrics* 2011; 127: e312-e18.	Did not report association between adiposity and cIMT
Shashaj B, Bedogni G, Graziani MP, Tozzi AE, DiCorpo ML, Morano D, et al. Origin of cardiovascular risk in overweight preschool children: A cohort study of cardiometabolic risk factors at the onset of obesity. JAMA Pediatrics. 2014;168(10):917-24.	Clinic population
Silva LR, Cavaglieri Cu, Lopes WA, Pizzi J, Coelho-e-Silva MJC, Leite N. Endothelial wall thickness, cardiorespiratory fitness and inflammatory markers in obese and non-obese adolescents. Brazilian Journal of Physical Therapy / Revista Brasileira de Fisioterapia. 2014;18(1):47-55.	Sample size <100
Skilton MR, Sullivan TR, Ayer JG, Harmer JA, Toelle BG, Webb K, Marks GB, Celermajer DS. Carotid extra-medial thickness in childhood: Early life effects on the arterial adventitia. *Atherosclerosis* 2012; 222: 478-82.	cIMT not measured
Slyper AH, Rosenberg H, Kabra A, Weiss MJ, Blech B, Gensler S, et al. Early atherogenesis and visceral fat in obese adolescents. International Journal of Obesity. 2014;38(7):954-8.	Sample size <100
Soriano-Rodriguez P, Osiniri I, Grau-Cabrera P, Riera-Perez E, Prats-Puig A, Carbonell-Alferez M, Schneider S, Mora-Maruny C, De Zegher F, Ibanez L, Bassols J, Lopez-Bermejo A. Physiological concentrations of serum cortisol are related to vascular risk markers in prepubertal children. *Pediatric Research* 2010; 68: 452-55.	Sample size < 100
Stabouli S, Kotsis V, Karagianni C, Zakopoulos N, Konstantopoulos A. Blood pressure and carotid artery intima-media thickness in children and adolescents: The role of obesity. *Hellenic Journal of Cardiology* 2012; 53: 41-47.	Clinic population
Terzis ID, Papamichail C, Psaltopoulou T, Georgiopoulos GA, Lipsou N, Chatzidou S, Kontoyiannis D, Kollias G, Iacovidou N, Zakopoulos N, Alevizaki M, Stamatelopoulos KS. Long-term BMI changes since adolescence and markers of early and advanced subclinical atherosclerosis. *Obesity* 2012; 20: 414-20.	Sample size < 100; adiposity and cIMT not measured within 24 months
Toledo-Corral CM, Ventura EE, Hodis HN, Weigensberg MJ, Lane CJ, Li Y, Goran MI. Persistence of the metabolic syndrome and its influence on carotid artery intima media thickness in overweight Latino children. *Atherosclerosis* 2009; 206: 594-98.	Sample size < 100; did not report association between adiposity and cIMT

Reference	Main reason(s) for exclusion
Toledo-Corral CM, Davis JN, Alderete TL, Weigensberg MJ, Ayala CT, Li Y, et al. Subclinical atherosclerosis in latino youth: Progression of carotid intima-media thickness and its relationship to cardiometabolic risk factors. Journal of Pediatrics. 2011;158(6):935-40.	Sample size <100; outcome is cIMT progression
Toledo-Corral CM, Myers SJ, Li Y, Hodis HN, Goran MI, Weigensberg MJ. Blunted nocturnal cortisol rise is associated with higher carotid artery intima-media thickness (CIMT) in overweight African American and Latino youth. Psychoneuroendocrinology. 2013;38(9):1658-67.	Main exposure of interest is cortisol level
Urbina EM, Khoury PR, McCoy C, Daniels SR, Kimball TR, Dolan LM. Cardiac and Vascular Consequences of Pre-Hypertension in Youth. Journal of Clinical *Hypertension* 2011; 13: 332-42.	Clinic population; study participation dependent on the presence of a secondary comorbidity
Urbina EM, Dabelea D, D'Agostino RB, Jr., Shah AS, Dolan LM, Hamman RF, et al. Effect of type 1 diabetes on carotid structure and function in adolescents and young adults: the SEARCH CVD study. Diabetes Care. 2013;36(9):2597-9.	Clinic population; study participation dependent on the presence of a secondary comorbidity
van der Merwe MT. Obesity in childhood and adolescence. *South African Medical Journal* 2012; 102: 289.	Editorial report
Verçoza A, Baldisserotto M, de los Santos C, Poli-de-Figueiredo C, d'Avila D. Cardiovascular Risk Factors and Carotid Intima-Media Thickness in Asymptomatic Children. Pediatric Cardiology. 2009;30(8):1055-60.	Sample size <100
Vijayasarathi A, Goldberg SJ. Comparison of carotid intima-media thickness in pediatric patients with metabolic syndrome, heterozygous familial hyperlipidemia and normals. Journal of Lipids. 2014;2014(546863).	Study participation dependent on the presence of a secondary comorbidity
Wilders-Truschnig M, Mangge H, Lieners C, Gruber HJ, Mayer C, Marz W. IgG antibodies against food antigens are correlated with inflammation and intima media thickness in obese juveniles. Experimental and Clinical Endocrinology and Diabetes. 2008;116(4):241-5.	Clinic population; sample size <100
Woelfle J, Roth CL, Wunsch R, Reinehr T. Pregnancy-associated plasma protein A in obese children: Relationship to markers and risk factors of atherosclerosis and members of the IGF system. European Journal of Endocrinology. 2011;165(4):613-22.	Clinic population; participants in lifestyle intervention trial
Yilmazer MM, Tavli V, Carti OU, Mese T, Guven B, AydIn B, et al. Cardiovascular risk factors and noninvasive assessment of arterial structure and function in obese Turkish children. European Journal of Pediatrics. 2010;169(10):1241-8.	Clinic population

cIMT: carotid intima-media thickness
